# Increased maternal leptin levels may be an indicator of subclinical hypothyroidism in a newborn

**DOI:** 10.5937/jomb0-32425

**Published:** 2022-04-08

**Authors:** Hande Karpuzoglu, Yasemin Ucal, Pinar Kumru, Murat Muhcu, Mustafa Eroglu, Muhittin Serdar, Mustafa Serteser, Aysel Ozpinar

**Affiliations:** 1 Acibadem Mehmet Ali Aydinlar University, School of Medicine, Department of Medical Biochemistry, Istanbul, Turkey; 2 Zeynep Kamil Research and Training Hospital, Department of Obstetrics and Gynecology, Istanbul, Turkey; 3 Umraniye Training and Research Hospital, Department of Obstetrics and Gynecology, Istanbul, Turkey; 4 Haydarpasa Numune Training and Research Hospital, Department of Obstetrics and Gynecology, Istanbul, Turkey

**Keywords:** eptin, congenital hypothyroidism, maternalfetal relations, newborn TSH (Thyroid Stimulating Hormone), maternal thyroid hormones, leptin, urođeni hipotiroidizam, odnosi majke i fetusa, TSH (hormon za stimulaciju štitne žlezde) novorođenčeta, hormoni štitne žlezde majke

## Abstract

**Background:**

Several factors may influence newborn thyroid-stimulating hormone (TSH) concentrations and cause subclinical hypothyroidism in a newborn. A sufficient level of leptin signalling is needed for the normal production of TSH and thyroid hormones by the thyroid gland. Our study aimed to investigate the correlation between maternal serum leptin concentration during the third trimester of pregnancy and newborn screening-TSH levels.

**Methods:**

This prospective cross-sectional study was conducted in obstetrics and gynaecology clinics of a state hospital between June and August 2013. Maternal venous blood samples were collected from 270 healthy pregnant women in the third trimester just before delivery. Measurements of maternal fT3, fT4, TSH, anti-thyroid peroxidase (TPO), and anti-thyroglobulin (anti-Tg) antibodies from serum samples were performed by chemiluminescence immunoassay. Maternal serum leptin levels were determined by ELISA. Dried capillary blood spots were used to measure newborn TSH levels.

**Results:**

Subjects were divided into two groups according to the neonatal TSH levels using a cut-point of 5.5 mIU/L. Median maternal serum leptin levels were significantly higher in newborns whose TSH levels were higher than >5.5 mIU/L [13.2 μg/L (1.3 - 46.5) vs 19.7 μg/L (2.4 - 48.5), p<0.05]. Serum leptin levels showed a negative correlation with maternal fT4 (r=0.32, p<0.05), fT3 (r=0.23, p<0.05), and a positive correlation with BMI (r=0.30, p<0.05).

**Conclusions:**

Our results suggest that high leptin levels in the third trimester of pregnancy influence maternal thyroid functions and might cause an increase in newborn TSH levels. Detection of high maternal serum leptin levels may be a reason for subclinical hypothyroidism.

## Abstract (sr)

**Uvod:**

Postoji nekoliko faktora koji mogu uticati na koncentraciju tiroidno-stimulišućeg hormona (TSH) kod novo - rođenčadi i na izazivanje subkliničkog hipotiroidizma. Da bi štitna žlezda normalno proizvodila TSH i tiroidne hormone potreban je dovoljan nivo signalizacije leptina. U našoj studiji smo želeli da istra'imo korelaciju između koncentracije serumskog majčinskog leptina tokom trećeg trimestra trudnoće i nivo TSH na skriningu kod novo rođenčadi.

**Metode:**

Ova prospektivna studija preseka sprovedena je na akušersko-ginekološkim klinikama jedne dr'avne bolnice u periodu od juna do avgusta 2013. Uzorci venske krvi majki prikupljeni su od 270 zdravih trudnica u trećem tromesečju neposredno pre porođaja. Merenje majčinskih fT3, fT4, TSH, antitireoidnih peroksidaza (TPO) i antitireoglobulinskih (anti-Tg) antitela iz serumskih uzoraka izvedeno je hemiluminiscentnim imunološkim testom. Nivo leptina u serumu kod majki je određen ELISA testom. Za merenje nivoa TSH kod novorođenčadi korišćene su suve kapilarne krvne tačke.

**Rezultati:**

Ispitanici su podeljeni u dve grupe prema neonatalnim nivoima TSH sa graničnom vrednošću od 5,5 mIU/L. Srednji nivo serumskog leptina je bio značajno veći kod novorođenčadi čiji su nivoi TSH bili veći od > 5,5 mIU/L [13,2 mg/L (1,3-46,5) naspram 19,7 mg/L (2,4-48,5), p < 0,05]. Nivo leptina u serumu je pokazao negativnu korelaciju sa majčinim fT4 (r = 0,32, p < 0,05), fT3 (r = 0,23, p < 0,05) i pozitivnu korelaciju sa BMI (r = 0,30 p < 0,05).

**Zaključak:**

Naši rezultati ukazuju da visoki nivoi leptina u trećem tromesečju trudnoće utiču na funkcije štitne žlezde majke i mogu izazvati i povećati nivo TSH kod novo - rođenčadi. Otkrivanje visokog nivoa majčinskog leptina u serumu mo'e biti razlog za subklinički hipotiroidizam.

## Introduction

Adipose tissue is an active endocrine organ that secretes various bioactive hormones called adipokines with multiple metabolic, neuroendocrine, cardiovascular, and inflammatory functions [1]. Leptin is a hormone that is exclusively secreted by adipose tissue and encoded by the *ob *gene. The primary role of leptin is to regulate energy homeostasis and suppress food intake, thereby inducing weight loss [2]. In addition, leptin has a vital role in the regulation and synthesis of thyroid hormones. In the paraventricular nucleus of the hypothalamus, leptin has a regulatory role in the expression and secretion of thyrotropinreleasing hormone (TRH); thus, it has a regulatory effect on thyroid-stimulating hormone (TSH) and thyroid hormone production [3].

One of the most common preventable causes of mental retardation among newborns is congenital hypothyroidism (CH). It is possible to diagnose newborn CH at early stages with newborn CH screening programs. Agenesis or dysgenesis of the thyroid gland and thyroid hormone production deficiency are the most common causes of permanent CH [4]. However, several other factors may influence newborn TSH concentrations and cause mild hypothyroidism. Iodine deficiency, maternal hypothyroidism [5], maternal medications, blocking antibodies [6], body mass index (BMI), and smoking [7], as well as weight gain during pregnancy [8], are well-known factors that may affect newborn thyroid function. A number of physiological and hormonal changes occur in pregnant women, such as changes in leptin concentrations. Increasing adipose tissue stores and secretion from the placenta results in elevated concentrations of leptin [9], which peaks at the end of the second or beginning of the third trimester and remains stable after that until delivery [10]. Leptin was suggested to play the primary role as a regulator of fetal growth and development [11].

The mechanisms of how leptin influences newborn thyroid function are not known. This study investigated the correlation between screening TSH levels in the newborn and serum leptin concentration of the mother at the third trimester to test whether increased leptin levels affect newborn thyroid function.

## Patients and Methods

### Patients

A total of three hundred healthy full-term pregnant women (37 - 41 weeks of gestational age) were included in this prospective cross-sectional study. This study did not include pregnant women with multiple gestations, abnormal ultrasound findings, and metabolic diseases. In addition, 30 out of 300 participants were excluded due to missing data (n=6), premature births (<37 weeks) (n=10), and thyroid hormone abnormalities during pregnancy (n=14). All infants were born by spontaneous delivery (n=270). There was no evidence of fetal distress during labour.

### Sample Collection and Laboratory Measurements

Maternal venous fasting blood (8 hours fasting) samples were collected in the 38^th^ week of pregnancy in the morning and were kept at -80 °C until analyses. Free T4 (fT4), free T3 (fT3), TSH, anti-thyroglobulin (anti-Tg) antibody, and anti-thyroid peroxidase (TPO) antibody measurements were made using chemiluminescence immunoassay (CLIA) with Advia Chemistry XPT System (Siemens Diagnostics, Germany). Enzyme-linked immunosorbent assay (ELISA) was used to measure leptin levels (DIA source Europe SA; Nivelles, Belgium). Inter-assay coefficient variation for fT4, fT3, TSH, anti-Tg antibody, and anti-TPO antibody at low levels were 1.21, 2.35, 2.28, 9.06, and 6.43%, and at high levels were 4.55, 1.61, 2.71, 8.14, and 1.74, respectively.

Heel-prick samples of whole blood were collected on filter paper cards from all newborns within 3 to 5 days after delivery. Dried capillary blood spots were used for TSH measurements.

### Statistical Analysis

Participants were divided into two groups according to newborn TSH levels using a cut-off value of 5.5 mIU/L based on the definitions of the Turkish national newborn screening program for congenital hypothyroidism [12]: group 1: TSH 5.5 mIU/L, group 2: TSH >5.5 mIU/L. Leptin levels and hormone concentrations showed a non-normal distribution; therefore, they are expressed as median and first and third quartiles, and intergroup comparisons were made using the Mann-Whitney U test. Pearson's correlation analysis was used to examine the correlation between newborn TSH and maternal leptin levels. Spearman's test was used to examine the correlations between leptin concentrations, thyroid hormones, and other clinical parameters in mothers. A p-value of <0.05 was considered statistically significant.

## Results

According to newborn TSH levels Table 1, compares demographical characteristics such as gestational and maternal age, body mass index, birth weight, and intrapartum laboratory findings. Gestational age, maternal age, body mass index, and birth weight were similar across the two groups (p>0.05). In addition, the groups showed similar findings for TSH, fT4, anti-TG, anti-TPO levels (p>0.05). On the other hand, maternal leptin levels were significantly higher [13.2 (1.3 - 46.5) vs 19.7 (2.4 - 48.5) μg/L, p<0.05] and fT3 levels were significantly lower [4.5 (3.3 - 5.9) vs 4.7 (3.5 - 6.36) pmol/L, p<0.05] in group 2 when compared to group 1 (Table 1).

**Table 1:** Demographic and laboratory characteristics of the studied population according to the newborn TSH levels. Maternal and newborn characteristics were compared with the newborn using a TSH cut-point of 5.5 mIU/L. Data arepresented as median, first and third interquertile ranges (IQR). p<0.05; Statistical significance of the difference between group I and II.

Newborn TSH (mIU/L)					
	Group I n=140<br>TSH 5.5 (mIU/L)		Group II n=130<br>>5.5 (mIU/L)		
	Median	2.5–97.5 P	Median	2.5–97.5 P	p-value
Maternal age	26	19–40	26	18.9–37.2	p>0.05
Pregnancy period	38	35–42	40	32–42	p>0.05
BMI	2 8 . 5	2 1 . 1 – 3 8 .2	2 8 . 4	2 1 . 2 – 3 9 . 2	p>0.05
Intrapartum TSH (mIU/L)	2.2	0.59–8.65	2.3	0.6–6.1	p>0.05
Intrapartum FT4 (pmol/L)	11.9	8.31–16.63	11.7	8.7–16.2	p>0.05
Intrapartum FT3 (pmol/L)	4.7	3.5–6.36	4.5	3.3–5.9	p^*^<0.05
Intrapartum anti TG (kIU/L)	15.8	10–122.6	26.8	10–86.2	p>0.05
Intrapartum anti TPO (kIU/L)	5.6	5–110	5.9	5–71.6	p>0.05
Intrapartum Leptin (μg/L)	13.2	1.3–46.5	19.7	2.4–48.5	p^*^<0.05
Baby Weight (gram)	3360	2042–4253	3280	2647–3923	p>0.05

**Figure 1:**
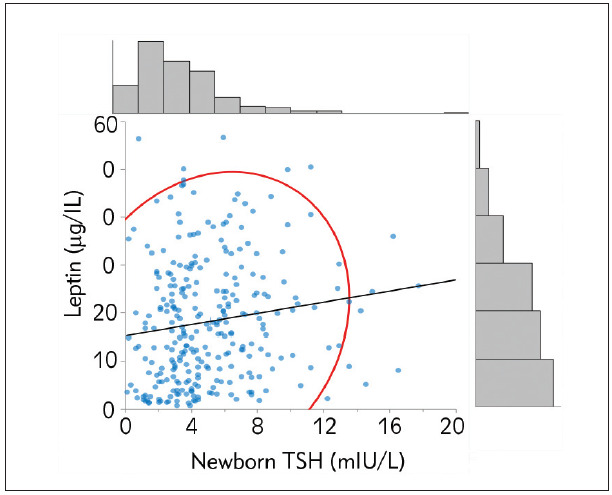
Correlation between maternal leptin and newborn TSH levels. Pearson’s correlation coefficient was determined as 0.16 (p<0.05). The frequency histogram shows the number of values (n) in the corresponding axis. The red curve represents 95% CI of the distribution.

**Figure 2:**
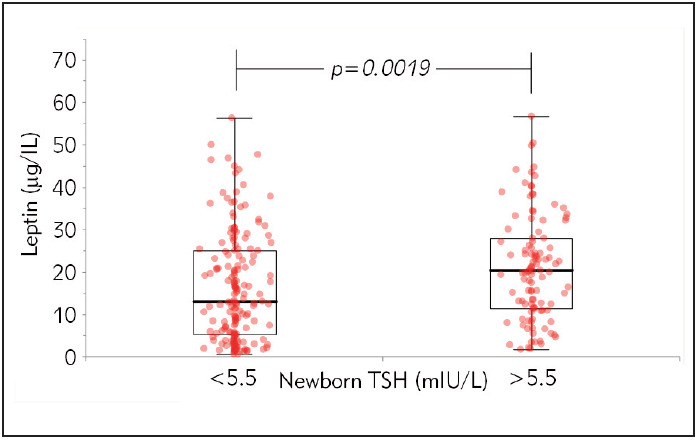
Relationship between newborn TSH and maternal serum leptin levels. p<0.05 was considered as a significant difference.

Correlations with leptin levels and other parameters are shown in Table 2. Serum leptin levels had negative correlations with intrapartum fT4, fT3, and positive correlations with BMI and newborn TSH levels. Figure 1 demonstrates the positive correlation between maternal serum leptin concentrations and newborn TSH levels (r=0.16, p<0.05) (Figure 2).

**Table 2:** Spearman’s *p* coefficients of correlations between maternal serum leptin levels and other parameters. Bolded values indicate statistical significance.<br>leptın as a novel placenta-derıved hormone ın humans

	Correlation (r)	*p*-value
Maternal age	-0.2	>0.05
Pregnancy period	0.014	>0.05
Intrapartum		
BMI	0.30	**<0.001**
TSH (mIU/L)	-0.03	>0.05
fT4 (pmol/L)	-0.32	**<0.001**
fT3 (pmol/L)	-0.23	<0.05
Anti-TG (kIU/L)	-0.049	**>0.05**
Anti-TPO (kIU/L)	-0.077	>0.05
Newborn		
Weight (kg)	0.049	>0.05
TSH (mIU/L)	0.16	**<0.05**

## Discussion

This study examined the relations between maternal serum leptin and thyroid hormone levels in the third trimester and TSH levels of the newborn measured from capillary blood samples and found a positive correlation between newborn TSH levels and maternal leptin levels. This is the first study to focus on the relation between third-trimester maternal serum leptin levels and the thyroid function of the newborn.

T3 and T4 circulating in the fetus are of maternal origin during the first trimester; whereas, developing the fetal thyroid gland increasingly contributes to the levels of the thyroid hormones from the beginning of the second trimester. Thyroid hormones have crucial roles in healthy fetal growth and development [13]. Several environmental factors may affect the thyroid function of the mother and the newborn [14]. Iodine deficiency [15], maternal thyroid hormones [5], low birth weight infants [16], pregnancy duration, maternal weight gain during pregnancy [8], high BMI, and lifetime smoking behaviour [7] are among the known factors associated with subclinical hypothyroidism in the newborn. One of the well-established causes of subclinical hypothyroidism during infancy is iodine deficiency; such that world Health Organization (WHO) recommended the use of a percentage of newborns with a TSH >5 mIU/L as a marker for population iodine deficiency [17]. The Turkish Newborn Screening Program recommends spot TSH levels of 5.5 mIU/L as a threshold for detecting congenital hypothyroidism [12] [15]. In our study, leptin levels were significantly higher in mothers of newborns whose TSH levels were higher than >5.5 mIU/L. Our results showed that increased maternal leptin levels could influence newborn TSH levels, which can be one of the main reasons for subclinical hypothyroidism in the newborn.

Leptin has modulatory roles in critical processes such as invasion, proliferation, protein synthesis, and placental cell apoptosis during early pregnancy [18]. In the later stages of a healthy pregnancy, increasing nutrient availability and regulating fetal growth is required. However, elevated leptin concentrations may represent a state of leptin resistance, which may be due to reduced bioactivity or reduced sensitivity at the hypothalamic level [19]. On the other hand, leptin overproduction by the placenta is associated with diabetes mellitus [20], hypertension [21], high BMI [7], and weight gain during pregnancy [8] [22]. Diabetes, obesity, and inflammation seem to be associated with the development of peripheral leptin resistance, which causes impaired leptin signalling in the brain [19]. Therefore, it is crucial to understand both the physiological and pathological effects of increased leptin levels during pregnancy on the mother and the newborn. In the present study, third-trimester leptin levels were positively correlated with BMI and maternal weight. These results are consistent with the findings of Sattar et al. [23], who found a positive correlation between BMI and third-trimester leptin levels. An increase in leptin levels is expected during pregnancy due to fat tissue accumulation [23].

Additionally, Shaarawy et al. [24] reported a positive correlation between weight gain and BMI as well as third-trimester leptin levels in pregnant women. However, in contrast with our findings, they did not find a significant difference between pregnant women with high and normal BMI in terms of leptin levels. The results of that study supported the suggestion that leptin release is mainly placenta-based during pregnancy [25].

Increased weight gain during pregnancy results in higher fetal weight gain [26]. Although leptin levels are known to increase with increasing fat tissue, we were not able to find a correlation between maternal leptin levels and the birthweight of the newborn. Similarly, Serapio et al. [27] found no correlation between maternal leptin levels and weight at birth. However, Manderson et al. [28] found an association between birth weight and cord leptin levels. On the other hand, Stefaniak et al. [29] found an association between birth weight and cord leptin levels (r=0.23; p=0.00), but not between the birthweight and maternal leptin levels. These studies support that cord leptin may increase fetal adipose tissue.

Various factors such as autoimmunity, fertility, hormones like estrogen, gender, insulin resistance, and high BMI affect the relationship between thyroid function and leptin [30]. The relation between leptin levels and thyroid function were examined in many studies. In our study, we found inverse relations between fT4/fT3 and leptin levels measured in the third trimester. However, we could not find correlations between maternal TSH hormone levels, levels of anti-TG and TPO, and leptin hormone. Pop et al. [22] recently examined the adverse effects of high BMI during pregnancy on thyroid function. In that study, pregnant women who gained much weight were found to have higher TSH levels and lower FT4 levels than pregnant women with a healthy weight increase. Their study speculated that the excessive leptin released from fat tissue might have affected the thyroid function of pregnant women [22]. Our results are in line with that study.

In the study by Iacobellis et al. [31], a positive correlation was found between TSH and leptin levels adjusted for BMI in euthyroid obese women (r=0.33 p=0.03). On the contrary, Betry et al. [32] showed a positive association between leptin and TSH levels independent of BMI in healthy individuals (p<0.001). Several studies showed conflicting results; some researchers showed a negative correlation, whereas others could not show a significant modulatory role for leptin on thyroid function [30].

In our study, the median leptin level was 19 μg/L (IQR: 1.546) in the third trimester, which was similar to the distribution of leptin levels in the study by Okdemir et al. [33]. In the cited study, the median leptin level was 7.32 μg/L (range, 1.00 - 33.19) and 12.54 μg/L (range, 1.07 - 45.75) in pregnant women with healthy and excess weight gain, respectively. On the other hand, Mazaki-Yovi's research found higher leptin levels at the third trimester: 30.2 μg/L (range, 16.9 - 43.5) [34]. These suggest that BMI and weight gain, as well as ethnicity, may affect leptin levels.

To the best of our knowledge, this is the first study revealing that maternal leptin levels may be correlated with maternal thyroid functions and increased newborn TSH levels and subclinical hypothyroidism. This study was limited in such a way that it was a cross-sectional study in which maternal blood was obtained only in the third trimester, and pregnancies were not regularly followed up. Larger prospective studies are warranted to elucidate the clinical relevance of our findings, focusing on the maternal thyroid functions, leptin levels, and weight gain during the first and second trimesters.

## Dodatak

### Funding

No funding sources available.

### Contributors

Aysel Ozpinar was the Principal Investigator of the study and contributed to the design of the research, data interpretation, and supported manuscript writing. Hande Karpuzoglu wrote the manuscript. Hande Karpuzoglu and Yasemin Ucal contributed to data analysis and interpretation. Pinar Kumru, Murat Muhcu, and Mustafa Eroglu were involved in subject selection and sample collection at the hospital. Muhittin Serdar contributed to the lab analysis and statistical analysis. Mustafa Serteser helped supervise the project. All authors drafted the manuscript, critically revised the manuscript, and agreed to be fully accountable for ensuring the integrity and accuracy of the work.

### Ethical approval

The study protocol was approved by the Acibadem University Ethics Committee (ATADEK 2013-507), and the study was conducted following the Declaration of Helsinki and its later amendments. All subjects gave informed consent before enrolment into the study.

### Consent to participate

All participants received informed consent, and a signed copy was filed.

### Consent for publication

All authors read and approved the final manuscript.

### Data availability

The data that support the findings of this study are available from the corresponding author upon reasonable request.

### Data deposition

The data will not be deposited.

### Geolocation information

Not available.

### Supplemental online material

Not available.

### Health and safety

Not available.

### Conflict of interest statement

All the authors declare that they have no conflict of interest in this work.
